# Implementation and evaluation of an elective quality improvement curriculum for preclinical students: a prospective controlled study

**DOI:** 10.1186/s12909-023-04047-0

**Published:** 2023-01-26

**Authors:** Jacqueline V. Aredo, Jack B. Ding, Cara H. Lai, Richard Trimble, Rebecca A. Bromley-Dulfano, Rita A. Popat, Lisa Shieh

**Affiliations:** 1 Stanford University School of Medicine, Stanford, CA USA; 2 Department of Medicine, University of California, San Francisco, 505 Parnassus Avenue, San Francisco, CA 94143 USA; 3 Adelaide Medical School, University of Adelaide, Adelaide, Australia; 4 Department of Epidemiology & Population Health, Stanford University School of Medicine, Stanford, CA USA; 5 Division of Hospital Medicine, Department of Medicine, Stanford University School of Medicine, Stanford, CA USA

**Keywords:** Quality improvement, Curriculum, Preclinical, Medical student, Physician assistant student

## Abstract

**Background:**

Quality improvement (QI) is a systematic approach to improving healthcare delivery with applications across all fields of medicine. However, exposure to QI is minimal in early medical education. We evaluated the effectiveness of an elective QI curriculum in teaching preclinical health professional students foundational QI concepts.

**Methods:**

This prospective controlled cohort study was conducted at a single academic institution. The elective QI curriculum consisted of web-based video didactics and exercises, supplemented with in-person classroom discussions. An optional hospital-based QI project was offered. Assessments included pre- and post-intervention surveys evaluating QI skills and beliefs and attitudes, quizzes, and Quality Improvement Knowledge Application Tool-Revised (QIKAT-R) cases. Within-group pre-post and between-group comparisons were performed using descriptive statistics.

**Results:**

Overall, 57 preclinical medical or physician assistant students participated under the QI curriculum group (*N* = 27) or control group (*N* = 30). Twenty-three (85%) curriculum students completed a QI project. Mean quiz scores were significantly improved in the curriculum group from pre- to post-assessment (Quiz 1: 2.0, *P* < 0.001; Quiz 2: 1.7, *P* = 0.002), and the mean differences significantly differed from those in the control group (Quiz 1: *P* < 0.001; Quiz 2: *P* = 0.010). QIKAT-R scores also significantly differed among the curriculum group versus controls (*P* = 0.012). In the curriculum group, students had improvements in their confidence with all 10 QI skills assessed, including 8 that were significantly improved from pre- to post-assessment, and 4 with significant between-group differences compared with controls. Students in both groups agreed that their medical education would be incomplete without a QI component and that they are likely to be involved in QI projects throughout their medical training and practice.

**Conclusions:**

The elective QI curriculum was effective in guiding preclinical students to develop their QI knowledge base and skillset. Preclinical students value QI as an integral component of their medical training. Future directions involve evaluating the impact of this curriculum on clinical clerkship performance and across other academic institutions.

**Supplementary Information:**

The online version contains supplementary material available at 10.1186/s12909-023-04047-0.

## Background

The prelude to the growing quality improvement (QI) culture in clinical medicine involves two landmark reports published by the U.S. Institute of Medicine, “To Err Is Human” in 2000 and “Crossing the Quality Chasm” in 2001 [1, 2]. These reports collectively highlighted medical errors as a topic of national concern and called for the implementation of QI and patient safety (QI/PS) efforts into medical practice and education. Clinical training programs have since integrated QI topics into their curricula, predominantly as a part of graduate medical education. However, the movement towards integrating QI education into undergraduate medical curricula has occurred at a slower pace. For example, one systematic review published in 2019 evaluated 18 studies of QI education to date targeted towards physician trainees, and found that 72% (*n* = 13) taught residents of various subspecialties whereas 28% (*n* = 5) focused on medical students [3]. Subsequently, an updated systematic review assessed 47 QI/PS education studies published between January 2019 and March 2022, and found that 81% (*n* = 38) were targeted towards graduate medial trainees and 19% (*n* = 9) towards undergraduate trainees [4]. Thus, despite the numerical increase in studies describing the development of QI curricula for physician trainees, the relative rate for medical students has lagged, possibly due to factors including a small evidence base to draw from and a lack of consensus guidelines for optimal curricular delivery.

Nonetheless, the importance of integrating QI concepts into medical training has been reinforced by both regulatory and licensing organizations globally. The Association of American Medical Colleges supports the incorporation of QI material into undergraduate medical education “across the continuum of physician professional development” [5]. The General Medical Council of the UK includes patient safety and quality improvement as a key component of professional values and behaviours that newly qualified doctors must demonstrate upon graduation [6]. The World Health Organization also advocates for the global integration of core patient safety topics throughout medical school training [7]. As a required component for medical licensing, QI topics are included in the United States Medical Licensing Examination (USMLE) step examinations with the expectation that medical students are receiving formal education and training on QI/PS [8]. Although the evidence for medical student-driven projects in improving patient outcomes remains limited, one study of various student-led quality interventions resulted in improved monitoring and outcomes for patients with diabetes mellitus in a community-based setting [9], suggesting that the benefits of early hands-on QI education may extend to patients in addition to learners.

Thus far, a majority of medical schools have targeted QI education towards third- or fourth-year clinical students [10–15], citing concerns about preclinical students’ abilities to grasp QI concepts in a clinical environment to which they have had little exposure [16]. Of the programs that have designed QI curricula for preclinical students [17–20], the curricula have been largely didactic- or workshop-based with limited clinical experiential components. Furthermore, the vast majority of these studies conducted pre-post curricular analyses that can only suggest that QI education for preclinical students may be effective in fostering their understanding of these healthcare concepts. Only one randomized controlled analysis has been performed which showed that a longitudinal QI curriculum was effective in improving core QI learning and certain domains of self-proficiency [20]; unfortunately, this module did not integrate well into the overall medical school curricula due to excessive time requirements, a common concern among educators [21]. These prior assessments of preclinical QI curricula have also largely involved in-person educational sessions. In the post-COVID-19 era, there is a need for online and blended QI learning opportunities that may not only be practical but also reach a broader audience of students [22]. One QI curriculum delivered to a small group of clinical medical students was conducted virtually which demonstrated improved pre-post knowledge scores and received positive feedback [23]. To our knowledge, the impact of online QI curricula on preclinical learner outcomes remains unexplored. Altogether, there remains a lack of standardization and accessibility of QI curricula designed for preclinical students to complete alongside a full academic workload.

Herein, we describe the development and implementation of a novel, predominantly web-based, elective QI curriculum for preclinical health professional students, which we offered concurrently with an optional hospital-based QI project. We evaluated the curriculum’s efficacy in teaching preclinical students foundational QI concepts by conducting a controlled analysis between a group of preclinical students who completed the QI curriculum and preclinical students who did not complete the curriculum but performed the study assessments.

## Methods

### Study design

**Fig. 1:**
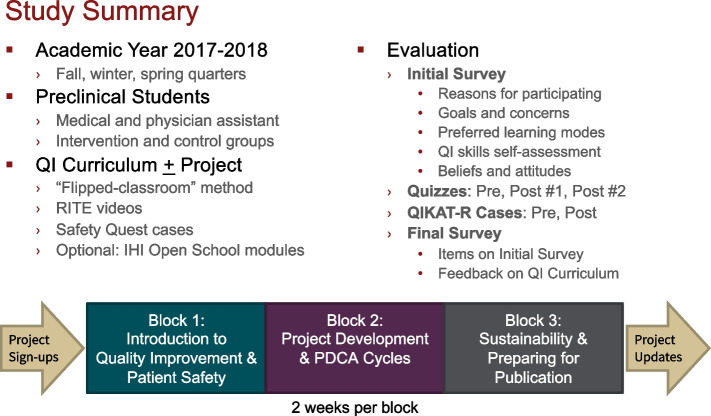
Study Summary. Abbreviations: *QI* quality improvement, *RITE* Realizing Improvement through Team Empowerment, *IHI* Institute for Healthcare Improvement, *QIKAT-R* Quality Improvement Knowledge Application Tool-Revised, *PDCA* Plan-Do-Check-Act

We conducted a prospective, controlled cohort study at Stanford University School of Medicine during the academic year 2017–2018 (Fig. 1). Preclinical medical (first- and second-year) and physician assistant (PA, first-year) students were invited to participate in a novel, elective QI curriculum which was offered over three iterations during the fall (September–December), winter (January–March), and spring (April–June) quarters. Students elected to complete the QI curriculum alone or in conjunction with a hospital-based QI project. Study controls were preclinical students who did not complete the QI curriculum but who completed the study assessments at the same time as the curriculum participants and who were compensated for their participation in the study. Study controls were recruited after the time period for inviting students to participate in the elective curriculum had ended, and represented a group of preclinical students who had not elected to participate. This project was evaluated by the institutional review board at Stanford University for determination of human subjects research and was determined to be exempt.

### QI curriculum

We developed the elective QI curriculum under the Kern medical education framework (Table S1) [24]. The curriculum incorporated the following publicly available web-based materials: Realizing Improvement through Team Empowerment (RITE) video tutorials [25], Safety Quest cases [26], and optional Institute for Healthcare Improvement (IHI) Open School modules [27] (Table S2). The QI curriculum was divided into three blocks, each spanning 2 weeks. A “flipped-classroom” approach was used for curriculum delivery whereby students asynchronously completed web-based assignments before engaging in in-person classroom discussions which occurred once during the second week of each block for a 50-minute session. The in-person sessions were led by a faculty member and study author with extensive experience in QI (LS) and were designed to review key concepts from the online content and the progress of ongoing QI projects. For the first two iterations of the curriculum (fall and winter quarters), attendance at these in-person sessions was optional. In the spring quarter, the curriculum was converted into a formal elective course for academic credit—based on the positive feedback received in the two quarters prior—thus beginning in the spring, attendance at the three in-person sessions became required.

In addition, all students participating in the QI curriculum were assigned a dedicated mentor—a project mentor for students participating in a QI project or a curriculum mentor for students completing the curriculum only—whom they could reach out to for guidance and questions. Each mentor was a faculty member or senior resident with experience in QI/PS and who felt comfortable teaching the curricular topics and supervising the respective project, if applicable.

### Measures

Study assessments consisted of initial and final surveys, quizzes, and Quality Improvement Knowledge Application Tool-Revised (QIKAT-R) cases [28] that were administered to students before and after the relevant topics in the curriculum were delivered (Table S1). Topics covered in the surveys are listed in Fig. 1. Questions on reasons for participating in the curriculum, goals, concerns, and aspects that students found valuable or that could be improved were open-ended and analyzed qualitatively. Under the Kern medical education framework [24], a targeted needs assessment was conducted as part of the initial survey which included eliciting preferred learning modes. At the conclusion of the curriculum, students were asked to rate the importance of the various educational activities for their learning.

QI knowledge was assessed though two sets of quizzes that covered content from the RITE videos, Safety Quest cases, and optional IHI modules. These quizzes were designed with input from the first and senior author, and pilot tested with all co-authors, who were preclinical students at the time of study design, prior to being administered to participants. Two sets of QIKAT-R cases, each consisting of three scenarios, were adapted to target understanding at the preclinical level. The cases were graded by two study authors (JA and CL), and the interrater reliability was evaluated using a two-way, mixed effects, absolute-agreement model for calculating the intraclass correlation coefficient [29]. Overall, the co-authors had moderate interrater reliability, with less agreement among the final case set (Table S3, Fig. S1).

### Blinding

Investigators were blinded to the identity and group status of students while evaluating their assessments. Each student was randomly assigned an identification number that was used to anonymize and track their study data.

### Statistical analyses

Between-group differences in categorical variables were assessed using Fisher’s exact test. Within-group pre-post comparisons of continuous variables were conducted using the paired t-test or Wilcoxon signed-rank test, whereas between-group comparisons utilized the unpaired t-test or Wilcoxon rank-sum test. For every continuous outcome variable, we reviewed whether the normality assumption was met; if it was, we used the paired or unpaired t-test for our analysis, and if it was not, we used the Wilcoxon signed-rank or Wilcoxon rank-sum non-parametric tests. Statistical significance was defined at a two-sided *P* < 0.05. All statistical analyses were performed using R version 4.0.2 (Vienna, Austria).

## Results

### Participants

Overall, 57 preclinical medical or PA students participated in the study (Table 1). Of these, 27 students participated in the elective QI curriculum, with medical (96.3%) and first-year (96.3%) students constituting the majority, and 23 chose to complete a QI project (Table S4). While most students reported having no prior experience in QI/PS, 29.6% of students joined with prior experience. Study controls consisted of a larger proportion of PA (36.7%) and second-year (56.7%) students, but a similar 30.0% with prior QI/PS experience.

**Table 1:** Baseline Characteristics of Participating Preclinical Health Professional Students

Characteristic	Curriculum ***N*** = 27	Control ***N*** = 30	P
Degree Program, N (%)			0.006
MD	26 (96.3)	19 (63.3)	
PA	1 (3.7)	11 (36.7)	
Year, N (%)			< 0.001
1st	26 (96.3)	13 (43.3)	
2nd	1 (3.7)	17 (56.7)	
Previous Experience in QI/PS, N (%)			1.000
No	19 (70.4)	21 (70.0)	
Yes	8 (29.6)	9 (30.0)	

*P* values were calculated between groups using Fisher’s exact test. Abbreviations: *N* sample size, *MD* Doctor of Medicine, *PA* physician assistant, *QI/PS* quality improvement/patient safety

Students cited a variety of reasons for curriculum participation (Table S5). Most reported a desire to explore a different aspect of healthcare that includes health systems, administration, and quality of care (*n* = 15). One student shared: “It’s an important facet of healthcare that we don’t really learn a lot about, and something to keep in mind while practicing.” Several students also expressed that the opportunity to complete a QI project was a primary motivating factor for participation (*n* = 7), including one student who stated that they wished to complete the curriculum only first and a project in the future.

Common goals that students noted for their participation in the curriculum included developing a greater understanding of healthcare QI processes (*n* = 14), attaining QI-related skills (*n* = 8), directly working on a QI project (*n* = 7), and producing scholarly work (*n* = 3; Table S5). Some students also shared that they hoped the curriculum would serve as an opportunity to learn how to incorporate QI into their future careers (*n* = 4).

The time commitment that curricular participation would involve was the greatest concern (*n* = 10; Table S5). Lack of prior experience with QI (*n* = 3) and uncertainty surrounding the definition and feasibility of the QI project (*n* = 3) were also noted as concerns. Several students expressed that they had no concerns about participating in the curriculum (*n* = 8).

### Preferred learning modes

In the initial study survey, students participating in the QI curriculum were asked about their preferred learning modes for this elective (Table S6), which would be completed alongside a full preclinical course load. Students were asked to rate the importance of each learning activity on a Likert scale ranging from 1 to 5, with 1 being Not Important, 2 Less Important, 3 Neutral, 4 Important, and 5 Very Important. Overall, project participation (mean 4.3, standard deviation [SD] 0.6), case-based sessions (mean 4.2, SD 0.7), and regular meetings with the QI project/curriculum mentor received the highest average ratings across the student cohort, and on average were all considered to be Important. In addition to these modes, two other learning modes that were frequently rated as Important/Very Important included interactive workshops on specific topics (68%) and online readings/videos on course material (64%).

### Quizzes

Students who completed the QI curriculum achieved a mean difference in initial and final scores (each out of 10 points) for Quizzes #1 and #2 of 2.0 (95% confidence interval [CI] 1.4–2.7, *P* < 0.001) and 1.7 (95% CI 0.8–2.7, *P* < 0.001), respectively, compared with 0.0 (95% CI -0.6-0.6, *P* = 0.913) and 0.4 (95% CI -0.1-0.8, *P* = 0.101) among the control group (Table 2). There were significant between-group differences in mean score changes for both Quiz #1 (2.0, 95% CI 1.1–2.9, *P* < 0.001) and Quiz #2 (1.4, 95% CI 0.3–2.4, *P* = 0.010), indicating that the improvement in QI knowledge was specific to the curriculum group.

**Table 2:** Initial and Final Quality Improvement Quiz Scores

	Curriculum Group^**a**^				Control Group				Between Groups	
Quiz	Initial	Final	Mean Difference (95% CI)	P	Initial	Final	Mean Difference (95% CI)	P	Mean Difference (95% CI)	P
#1	6.2 (1.3)	8.2 (1.2)	2.0 (1.4–2.7)	< 0.001	5.6 (1.4)	5.6 (1.4)	0.0 (− 0.6–0.6)	0.913	2.0 (1.1–2.9)	< 0.001
#2	4.9 (1.7)	6.6 (2.0)	1.7 (0.8–2.7)	< 0.001	3.7 (1.3)	4.0 (1.4)	0.4 (−0.1–0.8)	0.102	1.4 (0.3–2.4)	0.010

Values are presented as mean (standard deviation). Each quiz was graded on a numerical scale out of a total of 10 points. *P* values were calculated using paired t-tests for within-group comparisons and unpaired t-tests for between-group comparisons. Abbreviation: *CI* confidence interval

^a^ Paired scores for the curriculum group were evaluated in 22 students who had complete pre-post data

### QI skills self-assessment and beliefs & attitudes

Students in the curriculum group had improvements in their confidence with all ten QI skills assessed, including eight that were significantly improved from pre- to post-curriculum, and four with significant between-group differences when compared with the control group (Table 3). Conversely, none of the ten QI skills were significantly improved from initial to final assessment among controls. Of note, the significant between-group differences occurred when assessing “hard” QI skills—i.e., performing a root-cause analysis, breaking down a healthcare quality problem into a fishbone diagram, developing a SMART (specific, measurable, achievable, relevant, and time-bound) aim, and implementing a PDCA (Plan-Do-Check-Act) cycle—that are unlikely to be learned elsewhere in the health professional school curricula.

**Table 3:** Quality Improvement Skills Self-Assessment

Statement	Curriculum Group^**a**^			Control Group			Overall
*“I am confident in my ability to…”*	Initial	Final	P	Initial	Final	P	P
1. identify a QI need	3 [1–5]	4 [3–5]	0.012	4 [1–5]	4 [1–5]	0.480	0.089
2. perform a root-cause analysis	2 [1–4]	4 [2–4]	0.001	2 [1–4]	2 [1–5]	0.050	< 0.001
3. break down a healthcare quality problem into a fishbone diagram	2 [1–5]	4 [1–5]	< 0.001	1.5 [1–4]	2 [1–4]	0.929	< 0.001
4. identify stakeholders after a QI need has been identified	2 [1–5]	4 [1–5]	0.005	2.5 [1–5]	3 [1–5]	0.478	0.054
5. develop a proposal to close a quality gap	2 [1–4]	4 [1–5]	0.011	2.5 [1–5]	3 [1–5]	0.491	0.069
6. develop a SMART aim	2 [1–4]	4 [2–5]	< 0.001	2 [1–5]	2 [1–5]	0.072	< 0.001
7. identify appropriate measures for solving a problem	3 [1–5]	4 [2–5]	0.164	4 [1–5]	4 [1–5]	0.131	0.585
8. create a data collection plan consistent with time and resource limitations	3 [1–5]	4 [1–5]	0.019	3 [1–5]	3 [1–5]	0.288	0.189
9. implement a PDCA cycle for a QI initiative	2 [1–4]	3 [1–4]	0.001	2 [1–5]	2 [1–5]	0.660	< 0.001
10. sustain a change over time	2 [1–4]	3 [1–5]	0.841	3 [1–5]	3 [1–5]	0.280	0.284

Values are presented as median [range]. Each statement was assessed on a Likert scale out of a total of 5 points. P values for pre-post comparisons within groups were calculated using the Wilcoxon signed-rank test; *P* values for between-group comparisons were calculated using the Wilcoxon rank-sum test. Scale: 1-Strongly Disagree, 2-Disagree, 3-Neutral, 4-Agree, 5-Strongly Agree. Select statements were adapted from Mookherjee et al. 2013. Abbreviations: *QI* quality improvement; *SMART* specific, measurable, achievable, relevant, time-bound; *PDCA* Plan-Do-Check-Act

^a^ Paired scores for the curriculum group were evaluated in 21 students who had complete pre-post data

At the conclusion of the QI curriculum, students on average Agreed with feeling confident about their ability to engage in eight out of the ten QI skills assessed, all of which they initially Disagreed with or felt Neutral about with respect to their confidence (Table 3). After the QI curriculum, students were Neutral in their confidence with the remaining two skills which included implementing a PDCA cycle for a QI initiative and sustaining a change over time, both of which could be considered more longitudinal skills that may need more time to develop before confidence can be established. Nonetheless, confidence in the ability to conduct the former significantly improved from Disagree to Neutral following the curriculum, indicating that the curriculum was effective in boosting overall confidence in this skill.

In evaluating QI-related beliefs and attitudes, students in both groups agreed that QI holds an important role in their medical training and practice (Table 4), with agreement generally observed across all five statements. In addition, more students within the curriculum group shifted their pre-post beliefs towards having a future involvement in QI—in projects when in practice and as a core component of their clinician careers—versus the control group.

**Table 4:** Quality Improvement-Related Beliefs and Attitudes

	Curriculum Group^**a**^			Control Group			Overall
Statement	Initial	Final	P	Initial	Final	P	P
1. My medical education would be incomplete without a QI component.	4 [3–5]	4 [2–5]	0.520	4 [2–5]	4 [2–5]	0.059	0.582
2. I am likely to be involved in QI/PS projects throughout my medical training.	4 [3–5]	4 [2–5]	0.256	4 [1–5]	4 [2–5]	1.000	0.257
3. I am likely to be involved in QI/PS projects when in practice.	4 [3–5]	4 [3–5]	0.057	4 [2–5]	4 [2–5]	0.080	0.006
4. QI/PS will be a core component of my career as a physician.	4 [3–5]	4 [2–5]	0.145	4 [2–5]	4 [1–5]	0.218	0.047
5. My medical school should require QI/PS as part of its curriculum.	4 [3–5]	4 [3–5]	0.644	4 [2–5]	4 [1–5]	0.007	0.262

Values presented as median [range]. Each statement was assessed on a Likert scale out of a total of 5 points. P values for pre-post comparisons within groups were calculated using the Wilcoxon signed-rank test; *P* values for between-group comparisons were calculated using the Wilcoxon rank-sum test. Scale: 1-Strongly Disagree, 2-Disagree, 3-Neutral, 4-Agree, 5-Strongly Agree. Select statements were adapted from Mookherjee et al., 2013. Abbreviations: *QI* quality improvement, *QI/PS* quality improvement/patient safety

^a^ Paired scores for the curriculum group were evaluated in 21 students who had complete pre-post data

### QIKAT-R cases

The initial mean QIKAT-R scores (out of 27 points) of the curriculum group (13.2, SD 2.8) and the control group (14.0, SD 3.2) were comparable, with the between-group difference being non-significant (− 0.8, 95% CI -2.5-0.9, *P* = 0.343; Table S7). Students in the control group had significantly reduced mean scores between the initial and final sets of cases (14.0 versus 11.4, *P* < 0.001), suggesting that the final cases were more difficult than the initial ones. Students in curriculum group maintained similar mean scores on the initial and final sets of cases (13.2 versus 13.0, *P* = 0.739), which constituted a significant pre-post difference when compared with controls (mean difference between groups: 2.3, 95% CI 0.5–4.1, *P* = 0.012).

### Feedback

Student feedback on the QI curriculum at the end of each academic quarter was positive overall (Table S8). Students commented on various aspects of the curriculum that they found valuable; among these, the RITE video tutorials (*n* = 9), the actualization of a QI project (*n* = 8), and guidance from project/curriculum mentors (*n* = 4) were highlighted as being especially valued. One student shared: “Liked the flipped classroom aspect of the course and how accessible the videos could be to anyone.” Two students reported that the Safety Quest cases were valuable. Three students commented positively on the optional IHI modules, but two expressed that timing was a consideration: “I really enjoyed the IHI stuff when I had time. If this were an actual course, it would be the most useful material to include” and “The IHI modules were great but take up more time than a lot of people are willing to devote to QI.”

The most common suggested improvements for the QI curriculum included reducing curricular requirements especially if it were to remain purely optional (*n* = 4), expediting electronic health record access for projects (*n* = 3), checking in with less responsive project mentors (n = 3), and converting the curriculum into a formal elective course for academic credit (*n* = 2; Table S8), which took place during the spring quarter.

In the final study survey, students who completed the QI curriculum were asked about which learning modes they found most important for their QI education (Table S9). The most highly rated learning activities included QI project participation (mean 4.5, SD 0.6), regular meetings with QI project mentor (mean 4.3, SD 0.6), and the RITE video tutorials (mean 3.9, SD 0.9), all of which received a mean average rating of Important. These three learning modes were also the most frequently rated as Important/Very Important (70, 78, and 65%, respectively). In contrast, students were ambivalent about the importance of the Safety Quest cases, optional IHI modules, and in-person check-ins as learning modes (all received a mean average rating of Neutral).

## Discussion

Healthcare QI is becoming an essential topic in medical education, with the licensing authorities of certain countries mandating it as a core competency for graduating physicians [30]. Implementing QI curricula at the preclinical level enables early exposure to QI principles, providing the contextual grounding needed for a quality-conscious mindset in clinical training. However, there exist few evidence-based approaches to effectively integrating QI education into the preclinical years. This study showcases our experience in the development and implementation of a novel elective QI curriculum for preclinical students which was effective in teaching foundational QI principles.

Our blended elective QI curriculum was innovative in that it used a virtual interface to deliver essential content and provided the opportunity for students to directly apply their knowledge to a real-world hospital-based QI project. Asynchronous learning and digital curricula are increasingly relevant in medical education especially following the COVID-19 pandemic [31]. Some benefits include increased flexibility and personalization of study schedules [32, 33]. One randomized controlled study showed that a virtual learning environment significantly improved medical student satisfaction/engagement and recall compared with traditional didactic teaching [34], which constitutes passive learning [35]. The flipped classroom strategy, whereby students review instructional content online on their own time and then come together for class time dedicated to student-centered learning activities [36], has been shown to significantly improve student performance and satisfaction in a preclinical medical education setting [37]. Our curriculum offered three in-person classroom discussions to supplement the web-based teaching modules for interested students as well as an optional hospital-based QI project as an experiential component to help bridge the discrepancy between QI theory and practice, an issue previously highlighted [30]. Given the time constraints within a busy preclinical educational curriculum, the in-person classroom sessions (initially) and QI project were purely optional; this was done deliberately to help promote flexibility with the elective and encourage student participation. After the elective became a formal course for academic credit, the in-person sessions became mandatory, but remained concise, few in number, and aimed to review high-yield topics for QI learning. This educational format was effective in that we identified significantly increased knowledge and self-rated proficiency among preclinical students who completed the QI curriculum versus controls.

In implementing the curriculum, our initial survey assessed student preferences regarding learning modes and activities that they expected to find most beneficial for their learning. We found that engaging in QI practice through case-based sessions and project participation were regarded as among the most important. Activities that involved passing learning, such as regular student group meetings, classroom lectures, and expert panels, were rated the lowest. Accordingly, we tailored the in-person curricular sessions to reviewing key QI principles, discussing individual QI projects, and sharing learning lessons from prior faculty-conducted QI cases. For delivering QI content, our QI curriculum utilized the online RITE video tutorials. The RITE videos were developed based on expert experience using a variety of proven QI methods [25]; for this preclinical curriculum, we selected topics that we regarded as the most fundamental and easy for students to grasp, and which are covered in the USMLE step examinations [8]. Although initially rated of neutral importance in the initial survey, these videos were among the most highly valued in the concluding survey and were positively commented on the most frequently out of all aspects of the curriculum. These findings are in line with a prior questionnaire the evaluated learning preferences for patient safety topics among medical students in Singapore [38]. The investigators found that internet-based learning and discussion of real-life near misses were the most favored among students who had a variety of learning styles. A separate survey of medical students at the University of North Carolina found that students preferred to learn about QI/PS through real-life examples of QI projects or mistakes and errors presented by physicians and by a QI project on real-life patients [39], aspects which we included in our curriculum. In addition, our analysis found that mentorship was highly valued on QI projects, and that insufficient mentoring by lack of responsiveness could be a genuine concern. Supervision and mentorship by faculty experienced in QI has similarly been iterated as important among prior student learners [30, 40].

One key finding of this study is that regardless of study group, health professional students recognized QI as being integral to their medical education and careers. More than half the preclinical students from both groups agreed that QI/PS would play at least some role in their medical training and practice. Where the curriculum was effective in enacting a change in beliefs and attitudes was in increasing the number of students who expressed that QI/PS would likely have a role in their future clinician careers. While our results do not show a change in medians for the statements (given that most agreed with the importance of QI at baseline), the overall distributions did significantly shift and may be better illustrated by examining the proportions of students who Agreed or Strongly Agreed with the two statements: 1) “I am likely to be involved in QI/PS projects when in practice” (curriculum group: 67% initial versus 86% final; control group: 77% initial versus 70% final), and 2) “QI/PS will be a core component of my career as a physician” (curriculum group: 52% initial versus 76% final; control group: 53% initial versus 53% final). Altogether, these data suggest that the curriculum’s early exposure to QI may spark sufficient interest in QI among preclinical students such that it is more likely to become incorporated into their future careers.

Our study is unique among prior assessments of QI curricula among preclinical students in its successful implementation of a blended mode of instruction—incorporating web-based materials and a hospital-based experiential project—and through its controlled analyses. Our findings of improved self-assessed confidence levels and overall positive learner experience is in line with Shah et al., who evaluated a peer-led QI curriculum delivered through five workshops each of one-to-two hour duration to medical and PA students, a majority of whom were preclinical in their training [18]. The authors recorded significantly improved pre-post measures of self-reported confidence to perform, comfort with teaching, and objective knowledge of learned QI skills amongst students who undertook the course. However, the authors noted that enrollment for these workshops is limited to only 20% of the student population in a given year; and despite the 16-student cap per workshop, students provided feedback requesting even smaller discussion groups to increase individual participation. Thus, accessibility with the workshops could pose an issue. Additionally, some commentators have postulated that the workshop model of instruction, while effective at constructing a theoretical framework for QI understanding, is not readily generalizable to a real-world clinical context [30]. Brown et al. similarly evaluated the efficacy of an extracurricular workshop among first-year medical students, which included a simulation exercise involving the generation of a proposal aiming to improve the quality of an aspect of their medical school education [17]. This curriculum similarly achieved significant pre-post improvements in QI knowledge and self-assessed comfort levels with QI tasks; however, students reported a desire to carry out their project ideas and pursue further opportunities in QI.

The implementation of QI through longitudinal curricula during the preclinical years has also been explored. Dumenco et al. evaluated a 17-month preclinical QI program which was integrated into the regular medical school curriculum [19]. This study demonstrated a significant pre-post improvement in QI knowledge and perceived knowledge/skills among the students that was durable through the end of the third year of medical school. The authors complemented these findings with a cross-sectional analysis at a single timepoint, and found that students who completed the curriculum showed significantly greater measures of knowledge as well as QI-oriented attitudes compared with controls who did not complete the curriculum. Ogrinc et al. conducted a randomized controlled study evaluating a 7-month QI curriculum through which first-year medical students who completed the curriculum achieved significantly higher QI knowledge scores compared with controls [20]. However, this study highlights the limitations that may arise with the longitudinal structure. Students and faculty both expressed concern about the time requirements needed to implement the module, such that further iterations were discontinued. Although the curriculum was implemented into a problem-based learning format within the classroom, students continued to feel a disconnect with the clinical relevance of the QI topics. In addition, sufficient faculty with QI/PS expertise and course space are needed to accommodate this teaching longitudinally [19], which may not always be feasible given the number of preclinical topics that health professional schools must already cover.

A limitation of this study is the lack of randomization, which resulted in some heterogeneity between study groups. However, all participants were pre-clerkship and early in their training; thus, these students were lacking in substantial clinical experience and additional QI/PS exposures that could impact the study outcomes. Importantly, the proportion of students holding prior QI experiences was well-balanced between groups. Furthermore, at our institution, preclinical MD and PA students take the same core anatomy and clinical skills development courses together, and QI education is not otherwise introduced through other aspects of the preclinical MD or PA curricula. Another limitation is the relatively small sample sizes of the study groups, though our analyses indicate that they were sufficiently powered to detect meaningful between-group differences. As this was an elective curriculum, not all Stanford preclinical students participated in the study, thus selection bias is possible, though somewhat accounted for by the inclusion of a control group of students. We hoped that by enabling flexibility in participation, students could engage in the QI curriculum at a time best suited to their schedules. Given that only four students elected to complete the QI curriculum only without a project, we are unable to assess the impact that this less immersive experience may have on educational outcomes. However, one student did go on to participate in a QI project in a subsequent quarter. Lastly, the long-term impact of this curriculum on clinical performance and subsequent training decisions remains unknown. Moving forward, we hope to assess the effect of this curriculum on clerkship performance, particularly in the aspects involving systems-based knowledge and patient safety.

## Conclusions

In summary, medical educators have refrained from introducing healthcare QI topics to preclinical students citing concerns for a lack of contextual understanding. Our study demonstrates that the introduction of a blended yet predominantly web-based, elective QI curriculum with an optional experiential project to preclinical students is well-received, effective, and relevant in the current healthcare climate. While the long-term impact of an early introduction to QI to health professional students remains to be elucidated, the present study is encouraging in that the curriculum brought to awareness the possibility of incorporating QI into students’ future careers and practices, thus furthering the goals set out by the Institute of Medicine [1, 2]. As part of a greater initiative to promote equity in early QI education, all electronic content of the curriculum will remain publicly available and freely accessible to health professional students across institutions to incorporate alongside their regular coursework.

## Supplementary Information

**Additional file 1.** Supplementary Materials include Fig. S1 and Table S1-S9.

## Data Availability

Deidentified data used and/or analysed during the current study are available from the corresponding author on reasonable request.

## Abbreviations

QI: quality improvement
QI/PS: quality improvement and patient safety
USMLE: United States Medical Licensing Examination
PA: physician assistant
RITE: Realizing Improvement through Team Empowerment
IHI: Institute for Healthcare Improvement
QIKAT-R: Quality Improvement Knowledge Application Tool-Revised
CI: confidence interval
SMART: specific, measurable, achievable, relevant, time-bound
PDCA: Plan-Do-Check-Act
SD: standard deviation
