# Supplementary material for: Implementation and evaluation of an elective quality improvement curriculum for preclinical students: a prospective controlled study
Source: BMC Med Educ. 2023 Jan 26;23:66. doi: 10.1186/s12909-023-04047-0 (PMC9879619; doi:10.1186/s12909-023-04047-0)
Supplement: Supplementary file 1 — Additional file 1. Supplementary Materials include Fig. S1 and Table S1-S9. [file 12909_2023_4047_MOESM1_ESM.pdf]

Supplementary Materials

Figure S1. Bland-Altman Plot for Interrater Reliability on the QIKAT-R Cases.

Table S1. Elective Quality Improvement Curriculum Syllabus.
Table S2. Publicly Available Web-Based Resources Incorporated into the Quality Improvement Curriculum.
Table S3. Interrater Reliability of the QIKAT-R Cases.
Table S4. List of Student Quality Improvement Projects.
Table S5. Reasons that Preclinical Students Provided for Participating in the Elective Quality Improvement Curriculum, Goals, and Concerns.
Table S6. Preferred Learning Modes of Students Participating in the Elective Quality Improvement Curriculum.
Table S7. Distribution of QIKAT-R Case Scores.
Table S8. Student Feedback on the Quality Improvement Curriculum.
Table S9. Importance of Learning Modes from Students who Completed the Elective Quality Improvement Curriculum.

Figure S1. Bland-Altman Plot for Interrater Reliability on the QIKAT-R Cases. The mean difference between the two raters across all QIKAT-R cases was -0.67 (95% confidence interval -3.75 – 2.41). Abbreviation: QIKAT-R Quality Improvement Knowledge Application Tool-Revised.

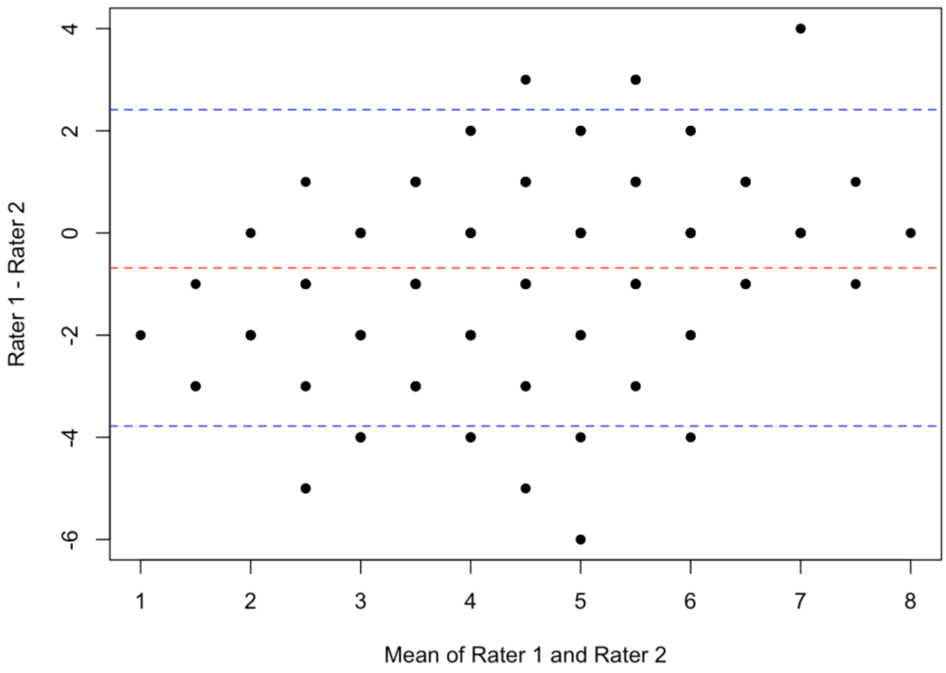

Table S1. Elective Quality Improvement Curriculum Syllabus.

**Session One: Introduction to Quality Improvement & Patient Safety**	
**Materials for Review**	**Assignments Due**
**RITE Videos**<br>#1: What is Quality?<br>#3: Life of a QI Project<br>#5: Current State<br>#6: Introduction to A3 Thinking<br>#7: SMART Goals<br><br>**Safety Quest Cases**<br>Case #1: Wrong Patient (Swiss Cheese)<br>Case #2: Adverse Event (Pareto)<br><br>**IHI Open School Modules (Optional)**<br>QI 101: Introduction to Health Care Improvement<br>PS 101: Introduction to Patient Safety	1) Baseline Survey<br>2) Online Pre-quiz
**Session Two: Project Development & PDCA Cycles**	
**Materials for Review**	**Assignment Due**
**RITE Videos**<br>#8: Understanding the Problem<br>#9: Metrics<br>#12: Developing Knowledge through Testing<br>#16: Communicating Progress and Results<br>#21: Processes<br><br>**Safety Quest Cases**<br>Case #9: Wrong Medication (Fishbone)<br>Case #4: Catheter Associated UTI (PDCA)<br><br>**IHI Open School Modules (Optional)**<br>QI 102: How to Improve with the Model for Improvement<br>QI 103: Testing and Measuring Changes with PDSA Cycles	1) Online Post-quiz #1
**Session Three: Sustainability & Preparing for Publication**	
**Materials for Review**	**Assignments Due**
**RITE Videos**<br>#22: Standardization<br>#26: Sustain Plans<br>#27: Reviewing Performance Data<br>#30: Closing an Improvement Project<br><br>**Safety Quest Cases**<br>Case #14: Breast Milk Mix-up (A3)<br>Case #20: Retained Sponge (Sustainability)<br><br>**IHI Open School Module (Optional)**<br>QI 104: Interpreting Data: Run Charts, Control Charts, and Other Measurement Tools<br>QI 105: Leading Quality Improvement	1) Online Post-quiz #2<br>2) Exit Survey<br><br>Students working on QI projects will be asked to provide updates on their projects!

Abbreviations: RITE Realizing Improvement through Team Empowerment, QI quality improvement, IHI Institute for Healthcare Improvement, PDCA Plan-Do-Check-Act.

Table S2. Publicly Available Web-Based Resources Incorporated into the Quality Improvement Curriculum.

Resource	Description	Website
RITE Videos	• Concise video tutorials produced by the Stanford Quality Improvement program in the Department of Radiology<br>• Focus is on concepts that are readily applicable to practice<br>• ~3-15 minutes per video	https://www.youtube.com/user/StanfordRadQuality/videos
Safety Quest Cases	• Interactive patient safety cases developed by the Stanford Quality Improvement program in the Department of Internal Medicine<br>• ~10-15 minutes per case	http://sm.stanford.edu/archive/safetyquest/
IHI Open School Modules (Optional)	• Provides a more in-depth background on concepts covered in each QI curriculum block<br>• Open School modules are used nation-wide to train healthcare professionals in QI<br>• Basic Certificate in Quality and Safety is available for those who complete the curriculum's optional modules in addition to a few more modules offered by the Open School<br>• ~30-60 minutes per case	https://my.ihi.org/topclass/lmsportal.aspx

Abbreviations: RITE Realizing Improvement through Team Empowerment, IHI Institute for Healthcare Improvement, QI quality improvement.

Table S3. Interrater Reliability of the QIKAT-R Cases.

QIKAT-R Case(s)	ICC (95% CI)
Case 1	0.58 (0.25-0.77)
Case 2	0.70 (0.48-0.83)
Case 3	0.78 (0.60-0.88)
Cases 1-3	0.70 (0.57-0.78)
Case 4	0.65 (0.40-0.80)
Case 5	0.51 (-0.24-0.78)
Case 6	0.42 (-0.33-0.74)
Cases 4-6	0.48 (0.09-0.68)
All Cases	
Cases 1-6	0.60 (0.41-0.72)

Interrater reliability was evaluated using a two-way, mixed effects, absolute-agreement model for calculating the ICC. Interrator reliability was assessed for each individual case, all three initial or final cases combined, and all six cases combined. Abbreviations: QIKAT-R Quality Improvement Knowledge Application Tool-Revised, ICC intraclass correlation coefficient, CI confidence interval.

Table S4. List of Student Quality Improvement Projects.

Topics
Factors contributing to extended inpatient length-of-stay
Identifying barriers to sepsis bundle compliance
Inappropriate antibiotic prescription for respiratory tract infections
Improving the delivery of value-based care
Reducing catheter-associated urinary tract infections
Decreasing unnecessary telemetry
Out-of-pocket healthcare costs
Improving communication with pediatric physicians at patient discharge
Impact of 3 Good Things on inpatient outcomes
Role of health literacy in patient understanding of hospital discharge instructions
Best practices with hospital interpreters

Table S5. Reasons that Preclinical Students Provided for Participating in the Elective Quality Improvement Curriculum, Goals, and Concerns.

Reasons for Participating	Goals	Concerns
Explore a different facet of healthcare	Get involved in a project, propose a change, evaluate the impact	Time commitment
Interested in healthcare systems and the impact on quality of care	Produce scholarly work: abstract/publications	Timeline for completing a project
Learn about QI through a structured curriculum	Develop skills in planning, strategy, business, negotiation	Coordinating with the project group
Obtain hands-on experience in QI	Understand root-cause analysis	Project definition
Learn the methodology and techniques used to study QI topics	Gain a broader toolset in understanding QI studies and practices	Current limited skillset
Work on a QI project from beginning to end	Learn about barriers to patient recovery	
Improve the patient experience from a systems perspective	Become familiar with study design and data analysis	
Complete the curriculum now, and a project in the future	"See whether it's a direction I would be interested in taking as a career path"	
Connect with faculty working on QI		
Opportunity for publications		
"Felt that this was an important part of medical education"		

Abbreviation: QI Quality Improvement.

Table S6. Preferred Learning Modes of Students Participating in the Elective Quality Improvement Curriculum.

**Statement**<br>*Please rate the importance of the following activities for your learning:*	**Average Rating (SD)**	**Rated Important/ Very Important, N (%)**
Online readings/videos on course material	3.5 (0.8)	16 (64)
Classroom lectures on course material	3.2 (1.0)	12 (48)
Small group discussion sessions	3.6 (1.0)	13 (52)
Case-based sessions	4.2 (0.7)	21 (84)
Interactive workshops on specific topics	3.8 (1.0)	17 (68)
Expert panels	3.4 (1.0)	11 (44)
Project participation	4.3 (0.6)	23 (92)
Regular meetings with QI project/curriculum mentor	4.0 (0.7)	19 (76)
Regular meetings with the student group as a whole	3.0 (0.8)	7 (28)

Each statement was assessed on a Likert scale out of a total of 5 points. Scale: 1-Not Important, 2-Less Important, 3-Neutral, 4-Important, 5-Very Important. Ratings are presented as mean (standard deviation) and the number and frequency of responses that were rated as Important or Very Important. Abbreviations: SD standard deviation, N number, % frequency. Data are presented from 25 students in the curriculum group who submitted complete data on these activities.

Table S7. Distribution of QIKAT-R Case Scores.

QIKAT-R Case(s)	Intervention Group	Control Group	Mean Difference Between Groups (95% CI)	P
Initial Cases				
Case 1	4.1 (1.3)	4.6 (1.6)	-0.4 (-1.2-0.4)	0.286
Case 2	4.2 (1.0)	4.2 (1.1)	0.0 (-0.6-0.6)	0.883
Case 3	4.8 (1.4)	5.3 (1.2)	-0.4 (4.8-5.3)	0.289
Cases 1-3	13.2 (2.8)	14.0 (3.2)	-0.8 (-2.5-0.9)	0.343
Final Cases				
Case 4	4.6 (1.3)	4.3 (1.0)	0.3 (-0.3-1.1)	0.286
Case 5	4.2 (1.0)	3.7 (1.2)	0.5 (-0.1-1.2)	0.083
Case 6	4.1 (1.2)	3.5 (1.1)	0.6 (-0.1-1.3)	0.074
Cases 4-6	13.0 (2.8)	11.4 (2.7)	1.5 (0.0-3.1)	0.053
Mean Difference Within Groups (95% CI; P)				
Cases (4-6) – (1-3)	0.2 (-1.6-1.2; P=0.739)	-2.6 (-3.8-[-1.3]; P<0.001)	2.3 (0.5-4.1)	0.012

Values are presented as mean (standard deviation). Each case was graded on a numerical scale out of a total of 9 points, with each set of initial or final cases out of 27 points. P values were calculated using paired t-tests for within-group comparisons and unpaired t-tests for between-group comparisons. Abbreviations: QIKAT-R Quality Improvement Knowledge Application Tool-Revised, CI confidence interval.

Table S8. Student Feedback on the Quality Improvement Curriculum.

Aspects that were Valuable	Aspects that could be Improved
RITE videos – explained the QI process well	Reducing requirements
Actualization of a QI project	Lectures on the material instead of videos
Connecting with responsive and excited project mentors	Different terminology used in different teaching modes
Safety Quest exercises	More disclosure of QI project as long-term
Structured curriculum and guidance	Require deliverables from groups in sessions
Learning how other projects were coming along	Safety Quest cases not as necessary
Optional IHI modules	Have a checklist of objectives separate from syllabus
Walking through the A3 in person	Check in with project mentors
Flipped classroom course design	Ensure EHR access as soon as possible
Quizzes for synthesizing the material	Host a final review of the most important QI concepts
Flexibility	Turn elective curriculum into a seminar/course

Abbreviations: RITE Realizing Improvement through Team Empowerment, QI quality improvement, IHI Institute for Healthcare Improvement, EHR electronic health record.

Table S9. Importance of Learning Modes from Students who Completed the Elective Quality Improvement Curriculum.

**Statement**<br>*Please rate how important the following activities were for your learning:*	**Average Rating (SD)**	**Rated Important/ Very Important, N (%)**
RITE Videos	3.9 (0.9)	15 (65)
Safety Quest Cases	3.2 (1.1)	9 (39)
Optional IHI Modules	3.0 (1.1)	5 (22)
In-person Check-ins	3.3 (0.9)	9 (39)
Project participation	4.5 (0.6)	16 (70)
Regular meetings with QI project mentor	4.3 (0.6)	18 (78)

Each statement was assessed on a Likert scale out of a total of 5 points. Scale: 1-Not Important, 2-Less Important, 3-Neutral, 4-Important, 5-Very Important. Ratings are presented as mean (standard deviation) and the number and frequency of responses that were rated as Important or Very Important. Abbreviations: SD standard deviation, N number, % frequency. Data are presented from 23 students in the curriculum group who submitted complete data on these activities.
